# Predicting success of oligomerized pool engineering (OPEN) for zinc finger target site sequences

**DOI:** 10.1186/1471-2105-11-543

**Published:** 2010-11-02

**Authors:** Jeffry D Sander, Deepak Reyon, Morgan L Maeder, Jonathan E Foley, Stacey Thibodeau-Beganny, Xiaohong Li, Maureen R Regan, Elizabeth J Dahlborg, Mathew J Goodwin, Fengli Fu, Daniel F Voytas, J Keith Joung, Drena Dobbs

**Affiliations:** 1 Molecular Pathology Unit, Center for Cancer Research, and Center for Computational and Integrative Biology, Massachusetts General Hospital, Charlestown, MA 02129, USA; 2 Department of Pathology, Harvard Medical School, Boston, MA 02115, USA; 3 Department of Genetics, Development and Cell Biology, Interdepartmental Graduate Program in Bioinformatics and Computational Biology, Iowa State University, Ames, IA 50011, USA; 4 Biological and Biomedical Sciences Program, Harvard Medical School, Boston, MA 02115, USA; 5 Department of Genetics, Cell Biology & Development, Center for Genome Engineering, University of Minnesota, Minneapolis, MN 55455, USA

## Abstract

**Background:**

Precise and efficient methods for gene targeting are critical for detailed functional analysis of genomes and regulatory networks and for potentially improving the efficacy and safety of gene therapies. Oligomerized Pool ENgineering (OPEN) is a recently developed method for engineering C2H2 zinc finger proteins (ZFPs) designed to bind specific DNA sequences with high affinity and specificity *in vivo*. Because generation of ZFPs using OPEN requires considerable effort, a computational method for identifying the sites in any given gene that are most likely to be successfully targeted by this method is desirable.

**Results:**

Analysis of the base composition of experimentally validated ZFP target sites identified important constraints on the DNA sequence space that can be effectively targeted using OPEN. Using alternate encodings to represent ZFP target sites, we implemented Naïve Bayes and Support Vector Machine classifiers capable of distinguishing "active" targets, i.e., ZFP binding sites that can be targeted with a high rate of success, from those that are "inactive" or poor targets for ZFPs generated using current OPEN technologies. When evaluated using leave-one-out cross-validation on a dataset of 135 experimentally validated ZFP target sites, the best Naïve Bayes classifier, designated ZiFOpT, achieved overall accuracy of 87% and specificity^+ ^of 90%, with an ROC AUC of 0.89. When challenged with a completely independent test set of 140 newly validated ZFP target sites, ZiFOpT performance was comparable in terms of overall accuracy (88%) and specificity^+ ^(92%), but with reduced ROC AUC (0.77). Users can rank potentially active ZFP target sites using a confidence score derived from the posterior probability returned by ZiFOpT.

**Conclusion:**

ZiFOpT, a machine learning classifier trained to identify DNA sequences amenable for targeting by OPEN-generated zinc finger arrays, can guide users to target sites that are most likely to function successfully *in vivo*, substantially reducing the experimental effort required. ZiFOpT is freely available and incorporated in the Zinc Finger Targeter web server (http://bindr.gdcb.iastate.edu/ZiFiT).

## Background

Zinc finger (ZF) DNA binding proteins can be used to target functional protein domains to specific regions in complex genomes. For example, zinc finger nucleases (ZFNs) have tremendous potential for introducing site-specific gene knockouts or gene targeting events with high efficiency in various cell types including human[1-3]. A ZFN consists of two zinc finger proteins (ZFPs) each fused to a monomeric *Fok*I nuclease domain. When the ZFPs co-locate to adjacent sequences within the genome, the nuclease monomers are able to dimerize, generating an active nuclease that cleaves the double-stranded DNA at the target site. In the presence of exogenous donor DNA, genetic material may be exchanged through repair by homologous recombination; alternatively, the break may be repaired by non-homologous end joining, which is an error-prone mechanism that commonly results in knockout mutations [4,5]. To date, ZFNs have been used to manipulate endogenous genes in several organisms, e.g., tobacco, maize, fruit fly, zebrafish, rats, and human [6-15], and are being evaluated in human clinical trials, including gene therapies to treat AIDS [16-18].

Zinc finger DNA binding domains, especially the C2H2 class of zinc fingers, have been exploited for performing targeted genome modification because they can be engineered to bind a wide range of desired DNA sequences. Each individual C2H2 zinc finger consists of an α-helix (the DNA "recognition helix") and a β-hairpin, stabilized by a single zinc ion coordinated through interactions with cysteine and histidine residues. Individual ZFs recognize and bind specific triplet DNA sequences through base-specific contacts within the major groove of double-stranded DNA[19]. Extended DNA sequences can be targeted by joining together several ZF domains [20,21].

ZFPs engineered using the recently developed Oligomerized Pool ENgineering (OPEN) method have been reported to function with high success rates *in vivo*, particularly for zinc finger nucleases (ZFNs) [8,9,15,20,22]. For constructing ZFPs that recognize 9-bp targets, the OPEN method involves combinatorial assembly and subsequent selection of fingers from three pre-constructed pools, each of which contains up to 95 different engineered ZF recognition helix "solutions" for a chosen DNA triplet [8,23]. Currently, pools are available for all 16 GNN triplets and several of the TNN triplets for each position in a three-finger array [8]. ZFNs generated by OPEN have been used to target genes in tobacco, zebrafish, and human cells with high efficiency [8-10].

Because using the OPEN procedure requires investment of time and effort and because there are often numerous potential targetable sites in any given gene, it is desirable to focus experiments on target sites that are most likely to yield functional ZFPs. For example, there are 315,186 OPEN ZFN sites in the protein encoding regions of the zebrafish genome (an average of 10.8 sites per transcript). While OPEN often generates ZFPs that function well in a bacterial two-hybrid (B2H) reporter system [8,9], it does not have a 100% success rate. Thus, to reduce the experimental effort involved in applying the OPEN procedure, we sought to develop a computational approach to identify the "best" targets, i.e., those most likely to be successfully targeted by OPEN, from among the relatively large number of theoretically "targetable" ZFP sites that may exist for any chosen gene or genomic region of interest.

In this study, we demonstrate that sequence characteristics of ZFP target sites, when used as input to Naïve Bayes or Support Vector Machine (SVM) classifiers, can be used to reliably predict whether a specific DNA sequence will (or will not) be successfully targeted by OPEN. The performance of these classifiers on two experimentally validated datasets of ZF target sites suggests that their use could substantially reduce the experimental effort required to generate a functional ZFN using the OPEN method.

## Results

Results from several groups [24-31] have suggested that ZFP recognition sites with a high purine nucleotide content, especially those containing several GNN-triplets, more frequently correspond to "active" targets for zinc finger proteins generated using modular assembly. To investigate whether such potential biases could be exploited to identify optimal sequences for ZFP targeting using OPEN, we analyzed sequence and base composition characteristics of sites targeted by this method.

For this study, we first generated an experimentally validated dataset, ZFTS135, consisting of 135 nine bp target sites for which OPEN did or did not successfully yield ZFPs. ZFTS135 includes 53 ZF target sites from recently published OPEN experiments [8,9] and 82 OPEN ZF target sites which we report here for the first time. Each target site in the dataset was assigned a class label of either "active" (79%) or "inactive" (21%). "Active" target sites were those yielding at least one ZFP that showed DNA-binding activity in a well-validated bacterial two-hybrid (B2H) reporter assay (defined as the ability to activate transcription by three-fold or more, a level previously shown to identify ZF arrays that possess high affinity and high specificity for their cognate DNA binding site [8,23]). "Inactive" target sites were those that failed to yield a ZFP that showed activity in the B2H reporter assay. All 135 functionally validated ZFP target sites and their assigned labels are provided in Additional File 1 - Table S1.

**Figure 1:**
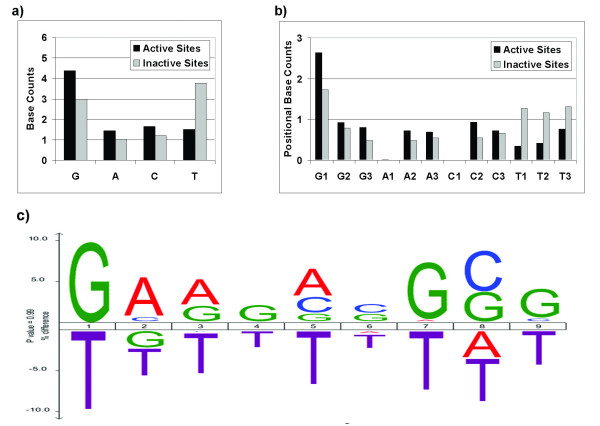
**Base composition differs in active versus inactive ZFP target sites**. **A) **Total *base counts *for active and inactive ZFP target sites (from ZFTS135, a dataset of 135 experimentally validated 9-bp target sites, see Additional File 1 - Table S1) reveal that variation in the average frequency of each base differentiates active versus inactive target sites. The total number of G and T residues relative to A and C is inflated because currently available OPEN pools are designed to target GNN and TNN triplets. **B) ***Positional base counts*, i.e., average base counts for each position within target site triplets (1^st^, 2^nd^, 3^rd^), suggest that thymine bases negatively impact ZFP binding at all three positions. **C**) An iceLogo [50] generated from ZFTS135 illustrates the difference in percentage composition of nucleotides at each position, from 1 - 9 (5' to 3'), between the positive class and the entire dataset. For example, 78% of all sites in ZFTS135 have a G in position 1, whereas 88% of all *active *sites have a G at position 1, resulting in a difference of 10%. Positive difference values indicate that, on average, the indicated bases are favored at those positions in active sites; negative difference values indicate that the indicated bases are disfavored. These position-specific differences in percentage composition also support the conclusion that thymine bases tend to occur in inactive targets (i.e., they have large negative propensities).

Figure 1 presents analyses of the sequence and base composition characteristics of ZFP target sites in the ZFTS135 dataset. The average number of times each base occurs in active and inactive targets is shown in Figure 1A. On average, active sites contain more guanines and fewer thymines than inactive targets. Because OPEN ZF finger pools are available exclusively for GNN and TNN triplet subsites at present, total guanine and thymine counts are inflated, compared to adenine and cytosine counts. To account for this, as well as the fact that specific bases, when located in different positions within a triplet subsite, may preferentially contact different amino acids, the average base occurrences were calculated for each position within the triplets (Figure 1B). This analysis identified thymine frequency, at any position within a triplet, as the primary difference between active and inactive target sites. Guanine, adenine, and cytosine typically appear more frequently in active sites than in inactive sites, compensating for the decrease in thymine content.

Differences in base composition at each position within active 9-bp target sites were also analyzed. As shown in Figure 1C, thymine is generally disfavored in active target sites, with strong negative propensities in the 1^st ^and 7^th ^positions of active target sites. Other residues showed marginally positive propensities in most positions. Because available OPEN reagents are currently limited to those that target GNN and TNN triplets [8] (and one ANN triplet; M. Maeder & J.K. Joung, unpublished data), it is not possible to evaluate the significance of the relatively low percentage of adenine and cytosine residues in positions 1, 4 and 7.

Taken together, the results of these analyses suggested that base composition biases in active versus inactive ZFP target sites could be exploited by machine learning classifiers to predict whether a specific DNA sequence can be targeted successfully using the OPEN procedure. Machine learning classifiers that use a string of sequence identities as input have been successfully applied to a variety of problems, including protein functional site classification [32-35]. Because several different machine learning classifiers we tested gave comparable results (data not shown), here we present representative results obtained using two types of classifiers: Naïve Bayes and support vector machines (SVMs).

We compared classifiers trained using three different target site sequence encodings: i) *sequence identity*: 9 nucleotide identities corresponding directly to the target site sequence; ii) *base counts*: 4 numerical values representing the overall base counts of G,A,C,T in the target site; iii) *positional base counts*: 12 numerical values encoding the position-specific base composition of the target site (see Methods for details).

Table 1 summarizes performance statistics for Naïve Bayes and SVM classifiers tested using the three different target site encodings and evaluated using leave-one-out cross-validation. In these experiments, classifiers were optimized for correlation coefficient, which is an indicator of how effectively a classifier identifies both positive (active) and negative (inactive) instances. All classifiers achieved correlation coefficients between 0.48 and 0.63, with accuracies ≥ 84%. For the practical application of identifying target sites for ZFPs that provide the greatest chance of success (for cases in which several potential target sites are available), it is appropriate to choose a classifier with a high specificity^+ ^value, i.e., one that predicts a smaller number of "active" sites with higher confidence, rather than a high correlation coefficient *per se*.

**Table 1:** Performance of classifiers in predicting active OPEN target sites

Classifier	Target site encoding	ROC AUC	Correlation Coefficient	Accuracy %	**Specificity**^**+ **^**%**	**Sensitivity**^**+ **^**%**
**Naïve Bayes**	**ZiFOpT** (Sequence Identity)	0.89	0.61	87	90	94
	Base Counts	0.79	0.57	87	89	94
	Positional Base Counts	0.84	0.59	87	88	97
**SVM**	Sequence Identity	0.76	0.48	84	86	95
	Base Counts	0.78	0.54	85	89	92
	Positional Base Counts	0.84	0.63	88	90	95

**Figure 2:**
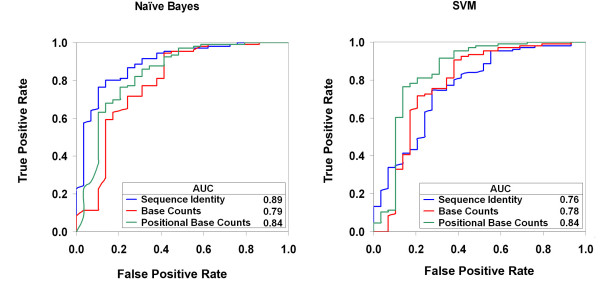
**Receiver Operating Characteristic (ROC) curves for Naïve Bayes and SVM classifiers**.

The receiver operating characteristic (ROC) curves in Figure 2 illustrate the tradeoffs between true positive rate (TPR), i.e., the percentage of active target sites *correctly *predicted as such, and false positive rate (FPR), i.e., the percentage of inactive sites *incorrectly *predicted to be active, for the different target sequence encodings. Using the base counts and positional base counts encodings, the Naïve Bayes and SVM classifiers gave similar results. Based on the Area Under the Curve (AUC) of the ROC curves, the best overall results were obtained using the sequence identity encoding with the Naïve Bayes classifier (AUC = 0.89), which slightly outperformed the best SVM classifier (AUC = 0.84). We designate the sequence-based Naïve Bayes classifier, ZiFOpT, for Zinc Finger OPEN Targeter.

To ensure that the performance of ZiFOpT on ZFTS135 was not over-estimated due to over-fitting, we generated a second completely independent data set of experimentally validated ZFP target sites. ZFTS140 consists of 140 9-bp target sites that were chosen by experts as ideal candidates for OPEN selection (see Additional File 2 - Table S2). Active ZFPs were found for 122 of the 140 sites tested. On this dataset, ZiFOpT performance was comparable in terms of overall accuracy (88%) and specificity^+ ^(92%), but with reduced ROC AUC (0.77). To assist users in choosing the best ZFP target sites, therefore, we also provide a confidence score derived from the posterior probability returned by ZiFOpT (see Methods), which allows users to rank the predicted active target sites. As shown in Table 2, choosing potential targets with confidence scores ≥ 6 (as opposed to scores < 6) results in improved accuracy (90% vs. 67%), specificity^+ ^(90% vs. 73%) and sensitivity^+ ^(100% vs. 85%).

**Table 2:** Performance of ZiFOpT on an independent test set (ZFTS140)

Confidence Score	Accuracy %	**Specificity**^**+ **^**%**	**Sensitivity**^**+ **^**%**
≥ 6	90	90	100
< 6	67	73	85

Due to the large number of potential OPEN target sites for most genomic targets of interest, it is desirable to identify a subset of target sites with the greatest chance of success. Currently, OPEN pools are available for 26 triplets in position 1, 21 triplets in position 2, and 23 triplets in position 3 of a 3-finger ZFP. Hence OPEN can, in theory, target 12,558 distinct sites. Because 415 of these sites are not targetable due to *dam *or *dcm *methylation, 12,143 distinct 9-bp ZFP target sites are currently targetable. The ZiFOpT classifier, when optimized for correlation coefficient, predicts that 8,412 (69%) of these sites will be active target sites. For ZF nuclease sites, which consist of two ZF array sites, OPEN can theoretically target a total 147,452,449 distinct nuclease sites (assuming a fixed number of nucleotides between the arrays). ZiFOpT predicts that only 70,761,744 (48%) of these nuclease sites will have two active sites.

An analysis of recently published OPEN ZFN sites in zebrafish [9] illustrates the value of ZiFOpT in reducing the experimental effort required to target a large number of genomic transcripts. In the previous study, at least one potential OPEN nuclease site was identified within the first three coding exons in ~86% of zebrafish transcripts [9]. As shown in Table 3, using a classification threshold that corresponds to a confidence score > 4 for the active sites (24% predicted FPR), ZiFOpT predicts that 15,565 (53%) of all zebrafish transcripts can be targeted *successfully *using OPEN. By restricting targets to those identified by ZiFOpT at a higher confidence score (> 8), the number of potential target sites for experimental testing could be reduced from 114,392 to 10,515, i.e., by ~ 90%. Thus, for functional genomic studies, ZiFOpT is a valuable tool for identifying sites most amenable to targeting by ZFNs. Indeed, we have used ZiFOpT to predict activity for all 315,186 OPEN ZFN targets previously identified in zebrafish [9]. These results are presented in Additional Files 3, 4, 5, 6, 7, 8, 9, 10, 11, 12, 13, 14, 15, 16, 17, 18, 19, 20, 21, 22, 23, 24, 25, 26 and 27.

**Table 3:** Summary of zebrafish OPEN ZFN target sites, classified by ZiFOpT

Confidence Score (Active Sites)	False Positive Rate^1 ^(FPR)	# of zebrafish transcripts targeted^2^		Average # of ZFN target sites^2 ^in transcripts containing nuclease sites	# of potential target sites^2 ^eliminated by using ZiFOpT	
**	**	25,174	(86%)	4.5	0	(0%)
> 4	24%	15,565	(53%)	2.3	78,934	(69%)
> 6	14%	12,622	(43%)	2.0	89,580	(78%)
> 8	7%	6,942	(24%)	1.5	103,877	(90%)

^1^estimated from training data ^2^in coding exons 1-3 **no classification

## Discussion

Detailed analyses of available high resolution structures for DNA-protein complexes support the conclusion that there is no simple general code for DNA-protein recognition [36]. For certain classes of DNA binding proteins, including the C2H2 zinc finger proteins, it may be possible to decipher some of the rules that govern protein-DNA recognition by exploiting the increasing availability of data regarding sequence determinants of binding affinity and specificity. For example, Stormo's group has utilized contact propensities and weight matrices to predict which target sites a zinc finger motif is most likely to bind [27,37]. Recently, Singh and colleagues utilized SVMs to predict whether a specific zinc finger protein will bind a specified target site [38]. Methods such as these utilize binding information for specific ZFPs interacting with a limited number of DNA target sites. In contrast, DNA microarray based experiments provide binding preferences of a transcription factor for thousands of potential sites [39-42]. These experiments should provide additional data for predicting and assessing transcription factor binding site models, including those for zinc finger proteins.

In the current study, we propose an approach for predicting whether a ZFP can be engineered to bind a specific DNA sequence without *a priori *knowledge of the ZFP amino acid sequence. We analyzed base composition features and position-specific base propensities in a dataset of 135 different DNA target sites for which the OPEN selection method had been experimentally attempted. Our goal was to use this information to develop a rapid and reliable machine learning classifier to identify DNA sequences most amenable to site-specific targeting by zinc finger arrays generated using the OPEN design procedure. Based on our results, we developed a server-based application, ZiFOpT, which implements a sequence identity-based Naïve Bayes classifier, and identifies active OPEN target sites with an estimated accuracy of 87% and ROC AUC of 0.89, when evaluated using cross-validation and optimized for correlation coefficient. ZiFOpT performance on an independent test set of 140 experimentally validated ZFP targets was lower in terms of AUC (0.77), as expected, due to the more challenging nature of this performance test. Importantly, confidence scores derived from posterior probabilities computed by ZiFOpT are provided for each predicted ZFP target site, allowing users to rank potential target sites and focus on those with the highest probability for success.

In our statistical analysis of active versus inactive target sites, we detected biases in position-specific base composition of ZF targets (Figure 1). Thus, we anticipated that classifiers in which we attempted to capture base count biases or position-specific base propensities in the sequence encoding might perform as well as those using sequence identity, particularly in light of the size of the dataset relative to the size of the feature space for the sequence identity representation. For the Naïve Bayes classifier, however, sequence identity outperformed positional base counts and gave the best overall performance, in terms of the AUC of the ROC curve (0.89). For the SVM classifier, using positional base counts as input did provide substantially better performance than sequence identity (0.84 vs. 0.76). Because the dataset used to train the SVM classifiers was smaller (to ensure a balanced number of positive and negative instances, see Methods), this difference in performance may be partly attributable to relatively sparse data for the sequence identity encoding.

Although the OPEN procedure tests only a small fraction of the total theoretical protein sequence space for the zinc finger recognition helix, it generates up to approximately 1 million ZFP combinations, clustered in what are expected to correspond to regions of optimal amino acid sequence space for the DNA target site of interest. Together with the results summarized in Figure 1, this suggests there are utilizable constraints on the DNA sequence space for 9-bp target sites that can be successfully targeted by ZFPs engineered by OPEN. For example, the results in Figures 1B and 1C indicate that increased thymine content in target sites, especially at positions 1 and 7, may preclude high affinity or high specificity binding. Previous studies have suggested that ZFP recognition sites with a relatively high purine nucleotide content are more often active targets for engineered zinc finger proteins [28,29]. These earlier conclusions were based on analysis of target sites containing predominantly GNN-triplets and for ZFPs generated using modular assembly. The current analysis confirms and quantifies the contributions of high purine content as an important determinant of success for sequences targeted using OPEN. More specifically, our analyses indicate that for three-finger ZFPs, it is advisable to avoid target sites containing many thymine bases.

Based on the results reported here, ZiFOpT will be valuable for guiding investigators using OPEN to ZFN target sites with the greatest opportunities for success. The calculations shown in Table 3 illustrate the potential reduction in experimental effort that could be achieved by using ZiFOpT to identify ZFP target sites for every protein encoded by the zebrafish genome. Also, ZiFOpT should be valuable for selecting targets among the 695,819 total OPEN nuclease targets identified in protein-encoding transcripts of the human genome (Ensemble V51.1) [D. Reyon and J. Sander, unpublished], and could assist investigators who wish to apply OPEN technology to target specific genes or genomic regions of interest in other organisms. ZiFOpT classifies potential target sites for OPEN-generated ZFPs as "active" or "inactive" and provides a confidence score for the prediction. ZiFOpT is freely available and incorporated in the Zinc Finger Targeter web server (http://bindr.gdcb.iastate.edu/ZiFiT) [43,44]. ZiFiT can scan a given DNA sequence of interest and identify every potential DNA site targetable by OPEN. With the integration of ZiFOpT, users will be able to evaluate the expected success rate of OPEN for target sites identified by ZiFiT.

## Conclusion

In this study, we developed machine learning classifiers that reliably identify DNA sites highly amenable to targeting by the OPEN zinc finger protein engineering method. Analysis of a dataset of 135 experimentally validated ZFP binding sites identified high thymine content as a significant barrier to effective targeting by OPEN. In addition, comparison of results obtained using three different target sequence encodings as input for Naïve Bayes and SVM classifiers suggested that positional context plays a significant role in ZFP target site recognition. Importantly, however, a simple encoding based on sequence identity is sufficient to identify the most promising ZFP target sites, with ~87% accuracy. As more ZFP functional data become available and we learn more about the sequence composition of fingers in OPEN pools, our predictions should improve. At present, the ZiFOpT classifier presented here is expected to reduce the experimental effort required to identify an active ZFP-target site pair by ~75%, compared with selection of target sites without classification. By restricting experimental targets to "active" OPEN sites predicted with highest confidence, experimental success rates should be significantly enhanced. This in turn should accelerate the application of zinc finger proteins as tools for precise genetic manipulation in basic genomics research as well as in gene therapy.

## Methods

### Definition of active and inactive ZFP target sites based on B2H assays

An *active *target site is a 9-bp DNA sequence for which the OPEN procedure has been used successfully to obtain at least one ZFP capable of binding the site with sufficient affinity and specificity to provide three-fold activation in a bacterial 2-hybrid (B2H) assay, i.e., to induce production of β-galactosidase by at least three-fold above the basal level of induction obtained using control constructs that lack the cognate ZFP target site [8,23,29]. An *inactive *target site is a 9-bp DNA sequence for which none of the corresponding OPEN-generated ZFPs tested were capable of producing a three-fold activation in the B2H assay.

### Datasets of experimentally validated ZFP-target sites

#### ZFTS135 (cross-validation dataset)

A zinc finger target site dataset generated from a group of 135 potential 9-bp zinc finger target sites (ZFTSs) that have been experimentally targeted using OPEN. For each ZFTS in the dataset, ZFPs have been selected using OPEN [8] and evaluated for DNA-binding activity *in vivo *using the B2H assay [10,23,29]. The sequences of all 135 ZFTS, together with their experimentally determined functional activity labels (active or inactive) are provided in Additional File 1 - Table S1. For 82 target sites in ZFTS135, functional activity labels, based on B2H assays, are reported here for the first time. The remaining 53 target sites, denoted by asterisks (*) were characterized previously [8,23,29] and experimental activity data were extracted from the Zinc Finger Database, ZiFDB (http://bindr.gdcb.iastate.edu/ZiFDB) [45].

#### ZFTS140 (independent test set)

This dataset is an independent group of 140 potential 9-bp ZFN target sites (none of which overlap with those in ZFTS135), which have been experimentally targeted using OPEN. These sites were chosen by experts in the field in order to generate a test set for rigorous evaluation of ZiFOpT performance. 122 (87%) of these sites were determined to be 'active' based on B2H assay results, as described above. The sequences of all 140 target sites, along with classification and confidence scores, are provided in Additional File 2 - Table S2.

### Machine learning classifiers

Naïve Bayes is a probabilistic classifier that assumes the independence of each attribute and generates models that are amenable to user interpretation, usually without compromising performance [46]. We used the implementation available in the WEKA package version 3.5.7 [47]. For each instance, the classifier returns a classification of either "active" or "inactive" based on the posterior probability (Bayes' rule). The value of the classification threshold (*θ*) can be selected based on the desired trade-off between sensitivity and specificity. We evaluated several classification performance measures (see below), using a standard leave-one-out cross validation procedure.

Support Vector Machines (SVMs) find a hyperplane in high-dimensional space that maximizes the distance between the different classes of data in that space. We implemented the SVM classifier using the wrapper class available for LIBSVM [48]. We tested several different kernel functions. Best results were obtained using the radial basis function (RBF) kernel. Optimal cost and gamma parameters were determined using a grid search algorithm. Because SVM classifiers are sensitive to the number of positive and negative instances in the training set, and because our dataset is unbalanced (106 positive and 29 negative instances), we used a variation of the standard leave-one-out cross validation technique. For each test case, we removed that instance and generated 10 randomized balanced training sets. The probability assigned to each test case was an average of the probability estimate generated from 10 randomized balanced training sets.

We also tested several other types of classifiers, including Decision Trees, and obtained results that were either comparable to or significantly worse than those obtained using ZiFOpT. Among the several Decision Tree algorithms we tested, the Logistic Model Tree (LMT) classifier performed the best with an AUC of ROC of 0.86.

### Target site sequence encoding

For each classifier, three different input sequence encodings were evaluated. The *sequence identity *input window consists of a target site represented as a 9 nucleotide DNA sequence, reading in the 5' to 3' direction on one strand (e.g., GTTGACGGC). The *base counts *input window consists of four single-digit values that represent the number of occurrences of each of the four DNA bases (G, A, C, T) within a target site (e.g., 4,1,2,2 for the target site in the preceding example). The *positional base counts *input window consists of a string of 12 values (3 sets of 4 digits), ranging from 0 to 3 and representing the number of times each base occurs in the first, second, and third positions within a triplet (e.g., 3,0,0,0;1,1,0,1;0,0,2,1, for the target site in the preceding example, in which G occurs in the first position of a triplet 3 times, once in the second and 0 times in the third.).

### Classification performance measures

We used several standard performance measures: *accuracy, correlation coefficient (CC), specificity^+^*, and *sensitivity*^+^, and the AUC for standard ROC curves as described by Baldi et al. [49]. Here *True Positives *(TP) is the number of validated targets correctly predicted to be "active" target sites, i.e., sites that have been targeted successfully by an OPEN-generated ZFP to produce > 3-fold activation in the B2H assay; *False Positives *(FP) is the number of "inactive" target sites incorrectly predicted to be "active" sites; *True Negative*s (TN) is the number of "inactive" target sites correctly predicted as such; *False Negatives *(FN) is the number of "active" target sites incorrectly predicted to be "inactive" sites.

$$\text{Accuracy}=\frac{TP+TN}{TP+FP+TN+FN}$$

$$\text{CC}=\frac{TP*TN-FP*FN}{\sqrt{\left(TP+FN)(TP+FP)(TN+FP)(TN+FN\right)}}$$

$${\text{Specificity}}^{+}=\frac{TP}{TP+FP}$$

$${\text{Sensitivity}}^{+}=\frac{TP}{TP+FN}$$

$$\text{False Positive Rate }\left(\text{FPR}\right)=\frac{FP}{FP+TN}$$

$$\text{True Positive Rate }(\text{TPR})=\frac{TP}{TP+FN}$$

A *Receiver Operating Characteristic (ROC) curve *displays the tradeoff between the true positive rate (hit rate) and the false positive rate (false alarm rate) for different discrimination thresholds [49]. The Area Under the Curve (AUC) of the ROC plot is valuable for comparing performance of different classifiers because it portrays the tradeoff between the false positive rate and the true positive over the range of classification threshold values.

### Confidence Score

The posterior probability returned by ZiFOpT for classifying each target site was used to generate a confidence score. Target sites with posterior probability were classified 'active' if they had posterior probability ≥ 0.5 and 'inactive' otherwise. For the 'active' class, the posterior probability was transformed to a scale from 0 to 9 by incrementing the confidence score by 1 as the posterior probability increased by 0.05 above 0.5. Therefore, a posterior probability of 0.75 corresponds to an 'active' classification with a confidence score of 5. For the 'inactive' class, the confidence score was incremented by 1 as the posterior probability decreased by 0.05 below 0.5. Therefore, a posterior probability of 0.25 corresponds to an 'inactive' classification with a confidence of 5.

## Competing interests

J.K.J. is an inventor on a patent application describing the OPEN method. The remaining author(s) declare that they have no competing interests.

## Authors' contributions

JS was responsible for experimental design, analysis of results, initial draft of manuscript, participated in discussions and manuscript revisions. DR parsed the data, ran the machine learning algorithms, participated in discussions and manuscript revisions. MM, ST, JF, XL, MRR, ED, MJG, JDS, and JK generated the experimental data and participated in manuscript reviews. FF, DD, DR, and DV participated in discussions, analysis of results, and manuscript revisions. All authors read and approved the final manuscript.

## Supplementary Material

Additional file 1 **ZFTS135 dataset**. Dataset of 135 nine base-pair zinc finger target sequences and activity labels used as the training set in this study Click here for file

Additional file 2 **ZFST140 dataset**. Dataset of 140 nine base-pair zinc finger target sequences, predictions, and actual activity label generated to validate the classifier. Click here for file

Additional file 3 **Zebrafish - chromosome 1 - classified ZFN target list**. Potential OPEN ZFN target sites in gene transcripts encoded on zebrafish chromosome 1 classified using ZiFOpT. Potential OPEN ZFN target sites in gene transcripts encoded on zebrafish chromosome 1. Gene ID and Transcript ID are from the Ensembl *Danio rerio *release 51 database. "Strand" indicates whether the "Target Site" shown (written 5' to 3') occurs on the forward (+) or reverse (-) strand. "ZFN Spacer Length" indicates the length of the spacer sequence located between the ZFN half-sites (5, 6, or 7 bps). "Coding Sequence Length" indicates the total nucleotide length of the coding sequence within the transcript and "ZFN Cleavage Site" indicates the nucleotide position of the cleavage site (i.e.-the first base of the "Target Site") within the coding sequence. Click here for file

Additional file 4 **Zebrafish - chromosome 2 - classified ZFN target list**. Potential OPEN ZFN target sites in gene transcripts encoded on zebrafish chromosome 2 classified using ZiFOpT. Data presented as described in the legend to Additional File 3 Click here for file

Additional file 5 **Zebrafish - chromosome 3 - classified ZFN target list**. Potential OPEN ZFN target sites in gene transcripts encoded on zebrafish chromosome 3 classified using ZiFOpT. Data presented as described in the legend to Additional File 3 Click here for file

Additional file 6 **Zebrafish - chromosome 4 - classified ZFN target list**. Potential OPEN ZFN target sites in gene transcripts encoded on zebrafish chromosome 4 classified using ZiFOpT. Data presented as described in the legend to Additional File 3 Click here for file

Additional file 7 **Zebrafish - chromosome 5 - classified ZFN target list**. Potential OPEN ZFN target sites in gene transcripts encoded on zebrafish chromosome 5 classified using ZiFOpT. Data presented as described in the legend to Additional File 3 Click here for file

Additional file 8 **Zebrafish - chromosome 6 - classified ZFN target list**. Potential OPEN ZFN target sites in gene transcripts encoded on zebrafish chromosome 6 classified using ZiFOpT. Data presented as described in the legend to Additional File 3 Click here for file

Additional file 9 **Zebrafish - chromosome 7 - classified ZFN target list**. Potential OPEN ZFN target sites in gene transcripts encoded on zebrafish chromosome 7 classified using ZiFOpT. Data presented as described in the legend to Additional File 3 Click here for file

Additional file 10 **Zebrafish - chromosome 8 - classified ZFN target list**. Potential OPEN ZFN target sites in gene transcripts encoded on zebrafish chromosome 8 classified using ZiFOpT. Data presented as described in the legend to Additional File 3 Click here for file

Additional file 11 **Zebrafish - chromosome 9 - classified ZFN target list**. Potential OPEN ZFN target sites in gene transcripts encoded on zebrafish chromosome 9 classified using ZiFOpT. Data presented as described in the legend to Additional File 3 Click here for file

Additional file 12 **Zebrafish - chromosome 10 - classified ZFN target list**. Potential OPEN ZFN target sites in gene transcripts encoded on zebrafish chromosome 10 classified using ZiFOpT. Data presented as described in the legend to Additional File 3 Click here for file

Additional file 13 **Zebrafish - chromosome 11 - classified ZFN target list**. Potential OPEN ZFN target sites in gene transcripts encoded on zebrafish chromosome 11 classified using ZiFOpT. Data presented as described in the legend to Additional File 3 Click here for file

Additional file 14 **Zebrafish - chromosome 12 - classified ZFN target list**. Potential OPEN ZFN target sites in gene transcripts encoded on zebrafish chromosome 12 classified using ZiFOpT. Data presented as described in the legend to Additional File 3 Click here for file

Additional file 15 **Zebrafish - chromosome 13 - classified ZFN target list**. Potential OPEN ZFN target sites in gene transcripts encoded on zebrafish chromosome 13 classified using ZiFOpT. Data presented as described in the legend to Additional File 3 Click here for file

Additional file 16 **Zebrafish - chromosome 14 - classified ZFN target list**. Potential OPEN ZFN target sites in gene transcripts encoded on zebrafish chromosome 14 classified using ZiFOpT. Data presented as described in the legend to Additional File 3 Click here for file

Additional file 17 **Zebrafish - chromosome 15 - classified ZFN target list**. Potential OPEN ZFN target sites in gene transcripts encoded on zebrafish chromosome 15 classified using ZiFOpT. Data presented as described in the legend to Additional File 3 Click here for file

Additional file 18 **Zebrafish - chromosome 16 - classified ZFN target list**. Potential OPEN ZFN target sites in gene transcripts encoded on zebrafish chromosome 16 classified using ZiFOpT. Data presented as described in the legend to Additional File 3 Click here for file

Additional file 19 **Zebrafish - chromosome 17 - classified ZFN target list**. Potential OPEN ZFN target sites in gene transcripts encoded on zebrafish chromosome 17 classified using ZiFOpT. Data presented as described in the legend to Additional File 3 Click here for file

Additional file 20 **Zebrafish - chromosome 18 - classified ZFN target list**. Potential OPEN ZFN target sites in gene transcripts encoded on zebrafish chromosome 18 classified using ZiFOpT. Data presented as described in the legend to Additional File 3 Click here for file

Additional file 21 **Zebrafish - chromosome 19 - classified ZFN target list**. Potential OPEN ZFN target sites in gene transcripts encoded on zebrafish chromosome 19 classified using ZiFOpT. Data presented as described in the legend to Additional File 3 Click here for file

Additional file 22 **Zebrafish - chromosome 20 - classified ZFN target list**. Potential OPEN ZFN target sites in gene transcripts encoded on zebrafish chromosome 20 classified using ZiFOpT. Data presented as described in the legend to Additional File 3 Click here for file

Additional file 23 **Zebrafish - chromosome 21 - classified ZFN target list**. Potential OPEN ZFN target sites in gene transcripts encoded on zebrafish chromosome 21 classified using ZiFOpT. Data presented as described in the legend to Additional File 3 Click here for file

Additional file 24 **Zebrafish - chromosome 22 - classified ZFN target list**. Potential OPEN ZFN target sites in gene transcripts encoded on zebrafish chromosome 22 classified using ZiFOpT. Data presented as described in the legend to Additional File 3 Click here for file

Additional file 25 **Zebrafish - chromosome 23 - classified ZFN target list**. Potential OPEN ZFN target sites in gene transcripts encoded on zebrafish chromosome 23 classified using ZiFOpT. Data presented as described in the legend to Additional File 3 Click here for file

Additional file 26 **Zebrafish - chromosome 24 - classified ZFN target list**. Potential OPEN ZFN target sites in gene transcripts encoded on zebrafish chromosome 24 classified using ZiFOpT. Data presented as described in the legend to Additional File 3 Click here for file

Additional file 27 **Zebrafish - chromosome 25 - classified ZFN target list**. Potential OPEN ZFN target sites in gene transcripts encoded on zebrafish chromosome 25 classified using ZiFOpT. Data presented as described in the legend to Additional File 3 Click here for file

## References

[B1] Carroll D Progress and prospects: zinc-finger nucleases as gene therapy agents Gene Ther 2008 15 22 1463 1468 10.1038/gt.2008.145 18784746 PMC2747807

[B2] Cathomen T Keith Joung J Zinc-finger nucleases: the next generation emerges Mol Ther 2008 16 7 1200 1207 10.1038/mt.2008.114 28178480

[B3] Urnov FD Rebar EJ Holmes MC Zhang HS Gregory PD Genome editing with engineered zinc finger nucleases Nat Rev Genet 2010 11 9 636 646 10.1038/nrg2842 20717154

[B4] Morton J Davis MW Jorgensen EM Carroll D Induction and repair of zinc-finger nuclease-targeted double-strand breaks in Caenorhabditis elegans somatic cells Proc Natl Acad Sci USA 2006 103 44 16370 16375 10.1073/pnas.0605633103 17060623 PMC1637589

[B5] Santiago Y Chan E Liu PQ Orlando S Zhang L Urnov FD Holmes MC Guschin D Waite A Miller JC Rebar EJ Gregory PD Klug A Collingwood TN Targeted gene knockout in mammalian cells by using engineered zinc-finger nucleases Proc Natl Acad Sci USA 2008 105 15 5809 5814 10.1073/pnas.0800940105 18359850 PMC2299223

[B6] Beumer K Bhattacharyya G Bibikova M Trautman JK Carroll D Efficient gene targeting in Drosophila with zinc-finger nucleases Genetics 2006 172 4 2391 2403 10.1534/genetics.105.052829 16452139 PMC1456366

[B7] Doyon Y McCammon JM Miller JC Faraji F Ngo C Katibah GE Amora R Hocking TD Zhang L Rebar EJ Gregory PD Urnov FD Amacher SL Heritable targeted gene disruption in zebrafish using designed zinc-finger nucleases Nat Biotechnol 2008 26 6 702 708 10.1038/nbt1409 18500334 PMC2674762

[B8] Maeder ML Thibodeau-Beganny S Osiak A Wright DA Anthony RM Eichtinger M Jiang T Foley JE Winfrey RJ Townsend JA Unger-Wallace E Sander JD Muller-Lerch F Fu F Pearlberg J Gobel C Dassie JP Pruett-Miller SM Porteus MH Sgroi DC Iafrate AJ Dobbs D McCray PB Jr Cathomen T Voytas DF Joung JK Rapid "open-source" engineering of customized zinc-finger nucleases for highly efficient gene modification Mol Cell 2008 31 2 294 301 10.1016/j.molcel.2008.06.016 18657511 PMC2535758

[B9] Foley JE Yeh JR Maeder ML Reyon D Sander JD Peterson RT Joung JK Rapid mutation of endogenous zebrafish genes using zinc finger nucleases made by Oligomerized Pool ENgineering (OPEN) PLoS ONE 2009 4 2 e4348 10.1371/journal.pone.0004348 19198653 PMC2634973

[B10] Townsend JA Wright DA Winfrey RJ Fu F Maeder M Joung JK Voytas DF High-frequency modification of plant genes using engineered zinc-finger nucleases Nature 2009 459 7245 442 445 19404258 10.1038/nature07845 PMC2743854

[B11] Lee HJ Kim E Kim JS Targeted chromosomal deletions in human cells using zinc finger nucleases Genome Res 2009 20 1 81 89 10.1101/gr.099747.109 19952142 PMC2798833

[B12] Shukla VK Doyon Y Miller JC DeKelver RC Moehle EA Worden SE Mitchell JC Arnold NL Gopalan S Meng X Choi VM Rock JM Wu YY Katibah GE Zhifang G McCaskill D Simpson MA Blakeslee B Greenwalt SA Butler HJ Hinkley SJ Zhang L Rebar EJ Gregory PD Urnov FD Precise genome modification in the crop species Zea mays using zinc-finger nucleases Nature 2009 459 7245 437 441 10.1038/nature07992 19404259

[B13] Voigt B Serikawa T Pluripotent stem cells and other technologies will eventually open the door for straightforward gene targeting in the rat Dis Model Mech 2009 2 7-8 341 343 10.1242/dmm.002824 19553695

[B14] Geurts AM Cost GJ Freyvert Y Zeitler B Miller JC Choi VM Jenkins SS Wood A Cui X Meng X Vincent A Lam S Michalkiewicz M Schilling R Foeckler J Kalloway S Weiler H Menoret S Anegon I Davis GD Zhang L Rebar EJ Gregory PD Urnov FD Jacob HJ Buelow R Knockout rats via embryo microinjection of zinc-finger nucleases Science 2009 325 5939 433 10.1126/science.1172447 19628861 PMC2831805

[B15] Zou J Maeder ML Mali P Pruett-Miller SM Thibodeau-Beganny S Chou BK Chen G Ye Z Park IH Daley GQ Porteus MH Joung JK Cheng L Gene targeting of a disease-related gene in human induced pluripotent stem and embryonic stem cells Cell Stem Cell 2009 5 1 97 110 10.1016/j.stem.2009.05.023 19540188 PMC2720132

[B16] Scott CT The zinc finger nuclease monopoly Nat Biotechnol 2005 23 8 915 918 10.1038/nbt0805-915 16082353

[B17] Kaiser J Gene therapy. Putting the fingers on gene repair Science 2005 310 5756 1894 1896 10.1126/science.310.5756.1894 16373552

[B18] Pearson H Protein engineering: The fate of fingers Nature 2008 455 7210 160 164 10.1038/455160a 18784697

[B19] Klug A The discovery of zinc fingers and their applications in gene regulation and genome manipulation Annu Rev Biochem 2010 79 213 231 10.1146/annurev-biochem-010909-095056 20192761

[B20] Sollu C Pars K Cornu TI Thibodeau-Beganny S Maeder ML Joung JK Heilbronn R Cathomen T Autonomous zinc-finger nuclease pairs for targeted chromosomal deletion Nucleic Acids Res 2010 20716517 10.1093/nar/gkq720 PMC3001086

[B21] Blancafort P Segal DJ Barbas CF Designing transcription factor architectures for drug discovery Mol Pharmacol 2004 66 6 1361 1371 10.1124/mol.104.002758 15340042

[B22] Zhang F Maeder ML Unger-Wallace E Hoshaw JP Reyon D Christian M Li X Pierick CJ Dobbs D Peterson T Joung JK Voytas DF High frequency targeted mutagenesis in Arabidopsis thaliana using zinc finger nucleases Proc Natl Acad Sci USA 2010 107 26 12028 12033 10.1073/pnas.0914991107 20508152 PMC2900673

[B23] Hurt JA Thibodeau SA Hirsh AS Pabo CO Joung JK Highly specific zinc finger proteins obtained by directed domain shuffling and cell-based selection Proc Natl Acad Sci USA 2003 100 21 12271 12276 10.1073/pnas.2135381100 14527993 PMC218748

[B24] Bae KH Kwon YD Shin HC Hwang MS Ryu EH Park KS Yang HY Lee DK Lee Y Park J Kwon HS Kim HW Yeh BI Lee HW Sohn SH Yoon J Seol W Kim JS Human zinc fingers as building blocks in the construction of artificial transcription factors Nat Biotechnol 2003 21 3 275 280 10.1038/nbt796 12592413

[B25] Carroll D Morton JJ Beumer KJ Segal DJ Design, construction and in vitro testing of zinc finger nucleases Nat Protoc 2006 1 3 1329 1341 10.1038/nprot.2006.231 17406419

[B26] Kim HJ Lee HJ Kim H Cho SW Kim JS Targeted genome editing in human cells with zinc finger nucleases constructed via modular assembly Genome Res 2009 19 7 1279 1288 10.1101/gr.089417.108 19470664 PMC2704428

[B27] Liu J Stormo GD Context-dependent DNA recognition code for C2H2 zinc-finger transcription factors Bioinformatics 2008 24 17 1850 1857 10.1093/bioinformatics/btn331 18586699 PMC2732218

[B28] Meng X Noyes MB Zhu LJ Lawson ND Wolfe SA Targeted gene inactivation in zebrafish using engineered zinc-finger nucleases Nat Biotechnol 2008 26 6 695 701 10.1038/nbt1398 18500337 PMC2502069

[B29] Ramirez CL Foley JE Wright DA Muller-Lerch F Rahman SH Cornu TI Winfrey RJ Sander JD Fu F Townsend JA Cathomen T Voytas DF Joung JK Unexpected failure rates for modular assembly of engineered zinc fingers Nat Methods 2008 5 5 374 375 10.1038/nmeth0508-374 18446154 PMC7880305

[B30] Sander JD Zaback P Joung JK Voytas DF Dobbs D An affinity-based scoring scheme for predicting DNA-binding activities of modularly assembled zinc-finger proteins Nucleic Acids Res 2009 37 2 506 515 10.1093/nar/gkn962 19056825 PMC2632909

[B31] Segal DJ Dreier B Beerli RR Barbas CF Toward controlling gene expression at will: selection and design of zinc finger domains recognizing each of the 5'-GNN-3' DNA target sequences Proc Natl Acad Sci USA 1999 96 6 2758 2763 10.1073/pnas.96.6.2758 10077584 PMC15842

[B32] Terribilini M Lee JH Yan C Jernigan RL Honavar V Dobbs D Prediction of RNA binding sites in proteins from amino acid sequence Rna 2006 12 8 1450 1462 10.1261/rna.2197306 16790841 PMC1524891

[B33] Narlikar L Hartemink AJ Sequence features of DNA binding sites reveal structural class of associated transcription factor Bioinformatics 2006 22 2 157 163 10.1093/bioinformatics/bti731 16267080

[B34] Capra JA Singh M Predicting functionally important residues from sequence conservation Bioinformatics 2007 23 15 1875 1882 10.1093/bioinformatics/btm270 17519246

[B35] Punta M Ofran Y The rough guide to in silico function prediction, or how to use sequence and structure information to predict protein function PLoS Comput Biol 2008 4 10 e1000160 10.1371/journal.pcbi.1000160 18974821 PMC2518264

[B36] Pabo CO Nekludova L Geometric analysis and comparison of protein-DNA interfaces: why is there no simple code for recognition? J Mol Biol 2000 301 3 597 624 10.1006/jmbi.2000.3918 10966773

[B37] Benos PV Lapedes AS Stormo GD Probabilistic code for DNA recognition by proteins of the EGR family J Mol Biol 2002 323 4 701 727 10.1016/S0022-2836(02)00917-8 12419259

[B38] Persikov AV Osada R Singh M Predicting DNA recognition by Cys2His2 zinc finger proteins Bioinformatics 2009 25 1 22 29 10.1093/bioinformatics/btn580 19008249 PMC2638941

[B39] Mukherjee S Berger MF Jona G Wang XS Muzzey D Snyder M Young RA Bulyk ML Rapid analysis of the DNA-binding specificities of transcription factors with DNA microarrays Nat Genet 2004 36 12 1331 1339 10.1038/ng1473 15543148 PMC2692596

[B40] Berger MF Philippakis AA Qureshi AM He FS Estep PW Bulyk ML Compact, universal DNA microarrays to comprehensively determine transcription-factor binding site specificities Nat Biotechnol 2006 24 11 1429 1435 10.1038/nbt1246 16998473 PMC4419707

[B41] Ragoussis J Field S Udalova IA Quantitative profiling of protein-DNA binding on microarrays Methods Mol Biol 2006 338 261 280 16888364 10.1385/1-59745-097-9:261

[B42] Berger MF Badis G Gehrke AR Talukder S Philippakis AA Pena-Castillo L Alleyne TM Mnaimneh S Botvinnik OB Chan ET Khalid F Zhang W Newburger D Jaeger SA Morris QD Bulyk ML Hughes TR Variation in homeodomain DNA binding revealed by high-resolution analysis of sequence preferences Cell 2008 133 7 1266 1276 10.1016/j.cell.2008.05.024 18585359 PMC2531161

[B43] Sander JD Maeder ML Reyon D Voytas DF Joung JK Dobbs D ZiFiT (Zinc Finger Targeter): an updated zinc finger engineering tool Nucleic Acids Res 2010 38 Suppl W462 468 10.1093/nar/gkq319 20435679 PMC2896148

[B44] Sander JD Zaback P Joung JK Voytas DF Dobbs D Zinc Finger Targeter (ZiFiT): an engineered zinc finger/target site design tool Nucleic Acids Res 2007 35 Web Server W599 605 10.1093/nar/gkm349 17526515 PMC1933188

[B45] Fu F Sander JD Maeder M Thibodeau-Beganny S Joung JK Dobbs D Miller L Voytas DF Zinc Finger Database (ZiFDB): a repository for information on C2H2 zinc fingers and engineered zinc-finger arrays Nucleic Acids Res 2009 37 Database D279 283 10.1093/nar/gkn606 18812396 PMC2686427

[B46] Buntine W Theory refinement on Bayesian networks Proceedings of the seventh conference (1991) on Uncertainty in artificial intelligence 1991 Los Angeles, California, United States: Morgan Kaufmann Publishers Inc

[B47] Witten IH Frank E Data mining: practical machine learning tools and techniques 2005 2 San Francisco: Morgan Kaufman

[B48] Chang CC Lin CJ LIBSVM: a library for support vector machines 2001

[B49] Baldi P Brunak S Chauvin Y Andersen CA Nielsen H Assessing the accuracy of prediction algorithms for classification: an overview Bioinformatics 2000 16 5 412 424 10.1093/bioinformatics/16.5.412 10871264

[B50] Colaert N Helsens K Martens L Vandekerckhove J Gevaert K Improved visualization of protein consensus sequences by iceLogo Nat Methods 2009 6 11 786 787 10.1038/nmeth1109-786 19876014

