# Supplementary material for: Predicting success of oligomerized pool engineering (OPEN) for zinc finger target site sequences
Source: BMC Bioinformatics. 2010 Nov 2;11:543. doi: 10.1186/1471-2105-11-543 (PMC3098093; doi:10.1186/1471-2105-11-543)
Supplement: Additional file 1 — ZFTS135 dataset. Dataset of 135 nine base-pair zinc finger target sequences and activity labels used as the training set in this study [file 1471-2105-11-543-S1.PDF]

Table S1: ZFTS135 dataset of zinc finger target sequences and activity labels.
DNA sequences of zinc finger target sites (shown reading 5' - 3') and functional activity labels for 135 experimentally validated ZFP-target site pairs analyzed in this study. "Active" indicates sites for which the OPEN method has been used to generate at least one corresponding zinc finger array that provides ≥ 3-fold activation of a β-galactosidase reporter gene in a bacterial 2-hybrid (B2H) reporter assay. For 82 target sites, B2H assay results are reported here for the first time. Asterisks (*) denote the remaining 53 target sites for which B2H activity results were reported previously and were extracted from ZiFDB (http://bindr.gdcb.iastate.edu/ZiFDB/).

Target 5' - 3'	Activity Label
GCATTTTTT	Inactive
GCCGCAGCT	Inactive
GCTTGAGCT	Inactive
*GGCGCCTAC	Inactive
*GGCGGAGAT	Inactive
*GGCGTTTGC	Inactive
GGGGAATAC	Inactive
GGTGTCTTT	Inactive
*GTGGCGGAT	Inactive
GTGTTTGAA	Inactive
GTTGATTTT	Inactive
GTTTTTGAG	Inactive
TAAGTTGTT	Inactive
TCTGCATTC	Inactive
*TCTGGCGCT	Inactive
*TCTGGTTTC	Inactive
TCTGTGTTC	Inactive
TCTTGGGTA	Inactive
*TGCGGCTGT	Inactive
TGCTGAGAC	Inactive
TGCTTTGTT	Inactive
TGGGTAGAA	Inactive
TGGTTTGTA	Inactive
TGTGTGTTC	Inactive
TTAGGAGGT	Inactive
TTTGAGGAT	Inactive
TTTGCTGAA	Inactive
TTTGTGGTG	Inactive
TTTGTGTGT	Inactive
*GAAGAAGCT	Active
*GAAGACGCT	Active
*GAAGATGGT	Active
*GAAGCAGCA	Active
*GAAGGATTC	Active
*GAAGTGGTC	Active
GAATTGGCG	Active
*GACGACGGC	Active
GACGCCGGA	Active
*GACGCTGCT	Active
GACTGAGAA	Active
GACTGGGCG	Active
GACTGGGCT	Active
GAGGGCGGC	Active
GAGGGGGCG	Active
*GAGTGAGGA	Active
GAGTGAGTT	Active
*GAGTTTGCC	Active
GATGAAGAC	Active
*GATGAAGCT	Active
GATGAGGCG	Active
GATGCCAAC	Active
GATGCGGCA	Active
GATGTAGCC	Active
GATTGAGTT	Active
GCAGAAGCT	Active
*GCAGCAGAG	Active
*GCAGCAGGA	Active
*GCAGCGGGC	Active
*GCAGGAGGT	Active
GCAGTGTGT	Active
GCCGAAGAT	Active
*GCCGCCGGC	Active
GCCGCTGGA	Active
*GCCGCTGGG	Active
*GCCGGCGGC	Active
GCCGGTGCA	Active
*GCCGGTGGC	Active
*GCCGTCGCC	Active
GCGGCCGCG	Active
*GCGGCGGAC	Active
*GCGGCTGGG	Active
GCGGGGGGC	Active
GCGGGTGTG	Active
GCGGTGGCG	Active
GCGTGGGCG	Active
GCGTTGGCG	Active
GCTGACTTT	Active
GCTGAGGCT	Active
*GCTGATGCC	Active
GCTGCAGAA	Active
GCTGCCGTC	Active
*GCTGCTGCC	Active
GCTGCTGGT	Active
GCTGGAGGG	Active
*GGAGGGGCT	Active
GGAGGTGAC	Active
GGAGGTGGA	Active
*GGAGGTGGT	Active
GGCGCAAAC	Active
*GGCGGCGGA	Active
GGCTGAGGC	Active
GGCTGGGCT	Active
GGCTGGGTG	Active
*GGGGAAGAG	Active
GGGGAAGAT	Active
*GGGGACGTC	Active
*GGGGAGGAG	Active
GGGGTCGAC	Active
GGGGTGGGT	Active
GGTGAATTT	Active
GGTGAGGCA	Active
GGTGCAGCA	Active
GGTGCTGAC	Active
GGTGGCGCT	Active
GGTGGGGTG	Active
GTAGAGGAG	Active
*GTAGATGGA	Active
GTAGCCTGT	Active
GTAGCTGGA	Active
GTCGACGCC	Active
*GTCGATGCC	Active
*GTCGGGGTA	Active
GTCTGAGGC	Active
GTCTGGGCT	Active
*GTGGACGCG	Active
*GTGGCTGGT	Active
*GTGTAGGGG	Active
TAAGCAGAA	Active
TAATTGGAG	Active
TCTGCTGGC	Active
TCTGGTGAG	Active
*TGGGAGTCT	Active
TGGGATGTT	Active
*TGGGGTGCC	Active
*TGGGTGGCA	Active
*TTAGAAGTG	Active

*GAGGACGGC	Active	GCAGCGGGA	Active	*TTATGGGAG	Active
*GAGGACGTG	Active	*GGAGGAGGT	Active	TTTGTTGGC	Active
*TGGGCTGCT	Active	GGAGGCGTG	Active	*GGTGCTGCC	Active
