# Supplementary material for: Predicting success of oligomerized pool engineering (OPEN) for zinc finger target site sequences
Source: BMC Bioinformatics. 2010 Nov 2;11:543. doi: 10.1186/1471-2105-11-543 (PMC3098093; doi:10.1186/1471-2105-11-543)
Supplement: Additional file 2 — ZFST140 dataset. Dataset of 140 nine base-pair zinc finger target sequences, predictions, and actual activity label generated to validate the classifier. [file 1471-2105-11-543-S2.PDF]

**Table S2:** ZFTS140 dataset of 140 experimentally validated zinc finger target site sequences, used as an independent ("blind") test set in this study. Target 5' - 3' = DNA sequence of zinc finger target site; Activity Label = actual activity, determined experimentally; Prediction: activity predicted by ZiFOpT; Confidence Score = confidence in prediction, ranging from 0 (lowest) to 9 (highest); see Methods for additional details.

| Target 5' - 3' | Activity Label | Prediction | Confidence Score |
|---|---|---|---|
| GGTGGAGCA | Active | Active | 9 |
| GGTGTCGAA | Active | Active | 9 |
| GTAGAAGAG | Active | Active | 9 |
| GTAGCAGTC | Active | Active | 9 |
| GTAGCTGCG | Active | Active | 9 |
| GTCGTTGCC | Active | Active | 9 |
| GTCTGAGTA | Active | Active | 9 |
| GTGGATGGT | Active | Active | 9 |
| GTGGCAGGA | Active | Active | 9 |
| GTGGCCGTG | Active | Active | 9 |
| TAATTGGGG | Active | Active | 9 |
| TCTGAGGAC | Active | Active | 9 |
| TCTGGTGAC | Active | Inactive | 9 |
| TGGGATGTG | Active | Active | 9 |
| TGGGCAGTG | Active | Active | 9 |
| TGGGGGGCA | Active | Active | 9 |
| TGGGTCGAC | Active | Inactive | 9 |
| TGTGACGGC | Active | Active | 9 |
| TGTGGGGGG | Active | Active | 9 |
| TTAGGGGAC | Active | Active | 9 |
| TGGGATGGA | Active | Active | 9 |
| GACGGCAAC | Active | Active | 9 |
| GTAGAGGGT | Active | Active | 9 |
| GCCGGAGAC | Active | Active | 9 |
| GCATGGGCA | Active | Active | 9 |
| GGTGATGCT | Active | Active | 9 |
| GCCGAAGAG | Active | Active | 9 |
| GACGGCTGT | Active | Active | 9 |
| GCTGCAGGT | Active | Active | 9 |
| GAGGATGTA | Active | Active | 9 |
| GCCGAAGTT | Active | Active | 9 |
| GACGGAGCT | Active | Active | 9 |
| GCTGATGGC | Active | Active | 9 |
| GCGGTTGCA | Active | Active | 9 |
| GACGGAGTC | Active | Active | 9 |
| GCAGGTGGA | Active | Active | 9 |
| GGGGAAGGT | Active | Active | 9 |
| GCCGCAGTG | Active | Active | 9 |
| GATGGTGAG | Active | Active | 9 |
| GGTTGGGAG | Active | Active | 9 |

| | | | |
|---|---|---|---|
| GCAGGCGCA | Active | Active | 9 |
| GAGGAGGGT | Active | Active | 9 |
| GGGGAAGGA | Active | Active | 9 |
| GAGGAGAAC | Active | Active | 9 |
| GGAGCCGGC | Active | Active | 9 |
| GCTGAGGGG | Active | Active | 9 |
| GCAGAAGTA | Active | Active | 9 |
| GAAGTAGCA | Active | Active | 9 |
| GCTGAAGCG | Active | Active | 9 |
| GATGATGGC | Active | Active | 9 |
| GAGGAAGCT | Active | Active | 9 |
| GTGGATGCA | Active | Active | 9 |
| GTGGCAGAA | Active | Active | 9 |
| TAAGAAGAG | Active | Active | 9 |
| GACGGAGGA | Active | Active | 9 |
| GATGAAGAA | Active | Active | 9 |
| GTAGCGGGT | Active | Active | 9 |
| GGTTAGGAT | Active | Active | 9 |
| GCGGCGGCC | Active | Active | 9 |
| GGTTGAGCG | Active | Active | 9 |
| GAGGAGGAG | Active | Active | 9 |
| GAGGCGTGT | Active | Active | 9 |
| GGAGGTGAG | Active | Active | 9 |
| GGAGGTGCC | Active | Active | 9 |
| GAAGAAGAG | Active | Active | 9 |
| GCGGCCGAA | Active | Active | 9 |
| GGAGAAGTA | Active | Active | 9 |
| GCTGAGGGC | Active | Active | 9 |
| GAGGACTGC | Active | Active | 9 |
| GGGGCTGCA | Active | Active | 9 |
| GAGGTAGTG | Active | Active | 9 |
| GAGGCGGAC | Active | Active | 9 |
| TGCGATGGA | Active | Active | 9 |
| GCTGGTGTC | Active | Active | 9 |
| TGGGCCGAC | Active | Active | 9 |
| GAGGCAGAA | Active | Active | 9 |
| GAAGCAGGC | Active | Active | 9 |
| GAGGATGGG | Active | Active | 9 |
| GCATGAGCT | Active | Active | 9 |
| GCTGGTGGC | Active | Active | 9 |
| GAGGCCTGT | Active | Active | 9 |
| GCTGCGGTG | Active | Active | 9 |
| GGAGGAGAT | Active | Active | 9 |
| GTGGTGGCT | Active | Active | 9 |
| GGATGAGCC | Active | Active | 9 |
| GCTGACTGC | Active | Active | 9 |
| GCGGGAGGG | Active | Active | 8 |
| GCGGTAGCT | Active | Active | 8 |

| | | | |
|---|---|---|---|
| GCTGACGGT | Active | Active | 8 |
| GCTGAGGAA | Active | Active | 8 |
| GCTGCAGAA | Active | Active | 8 |
| GCTGGTGAA | Active | Active | 8 |
| GCTGTCGAA | Active | Active | 8 |
| GCTGTTGGG | Active | Active | 8 |
| GGAGACGGT | Active | Active | 8 |
| GGCGACGGC | Active | Active | 8 |
| GGCGAGGAA | Active | Active | 8 |
| GGCGCAGGG | Active | Active | 8 |
| GGGGCAGTG | Active | Active | 8 |
| GGGGCGGGT | Active | Active | 8 |
| GGGGCTGAG | Active | Active | 8 |
| GGGGGAGGG | Active | Active | 8 |
| GGTGAAGAG | Active | Active | 8 |
| GGTGCCGAG | Active | Active | 8 |
| GACTTTGGT | Inactive | Active | 7 |
| GAGGCAGCA | Active | Active | 7 |
| GAGGCCGAG | Active | Active | 7 |
| GAGGCCGGC | Active | Active | 7 |
| GAGGGAGGA | Active | Active | 7 |
| GAGGTGGGT | Active | Active | 7 |
| GCAGCAGGG | Active | Active | 7 |
| GCAGGGGCG | Active | Active | 7 |
| GCAGGTGCT | Active | Active | 7 |
| GCCGCGGCC | Active | Active | 7 |
| GCGGCTGCC | Active | Active | 7 |
| GCGGCTGCG | Active | Active | 7 |
| GAAGGGTGC | Active | Active | 6 |
| GAAGGTGTT | Active | Active | 6 |
| GAAGTCTGC | Active | Active | 6 |
| GACGAAGGC | Active | Active | 6 |
| GACGACGAA | Active | Active | 6 |
| GAGGAGGTC | Active | Active | 6 |
| GTCGTGGCC | Inactive | Active | 5 |
| GTAGGAGAG | Inactive | Active | 5 |
| GTCGGCGTA | Inactive | Active | 5 |
| GGTGCTGCG | Inactive | Active | 5 |
| GAAGGGGCC | Active | Active | 5 |
| GCAGCCGCA | Inactive | Active | 4 |
| GGAGTTGTT | Inactive | Active | 4 |
| GTCTGAGCA | Inactive | Active | 4 |
| GGGTTTGCA | Inactive | Active | 4 |
| GGTGATGAA | Inactive | Active | 3 |
| GTCGCAGTA | Inactive | Active | 3 |
| GCTTAGGGT | Inactive | Active | 3 |
| GCGTTTGAG | Inactive | Active | 2 |
| GTCGCTGTC | Inactive | Active | 1 |

| | | | |
|---|---|---|---|
| TCTGGAGAT | Inactive | Inactive | 1 |
| TGTGAATGT | Inactive | Inactive | 1 |
| GGCGGAGCA | Inactive | Active | 1 |
| GGTTTTGAG | Inactive | Active | 0 |
